# Supplementary material for: Characterization and Preliminary Application of a Novel Lipoxygenase from Enterovibrio norvegicus
Source: Foods. 2022 Sep 15;11(18):2864. doi: 10.3390/foods11182864 (PMC9498203; doi:10.3390/foods11182864)
Supplement: Supplementary file 1 [file foods-11-02864-s001.zip › foods-1905314-supplementary.pdf]

Supplementary Materials

Characterization and Preliminary Application of a Novel Lipoxygenase from *Enterovibrio norvegicus*

Bingjie Zhang [1], Meirong Chen [1], Bingjie Xia [1], Zhaoxin Lu [1], Kuan Shiong Khoo [2], Pau Loke Show [2] and Fengxia Lu [1,*]

[1] College of Food Science and Technology, Nanjing Agricultural University, Nanjing 210095, China
[2] Faculty of Science and Engineering, University of Nottingham Malaysia, Jalan Broga 43500, Malaysia
* Correspondence: lufengxia@njau.edu.cn

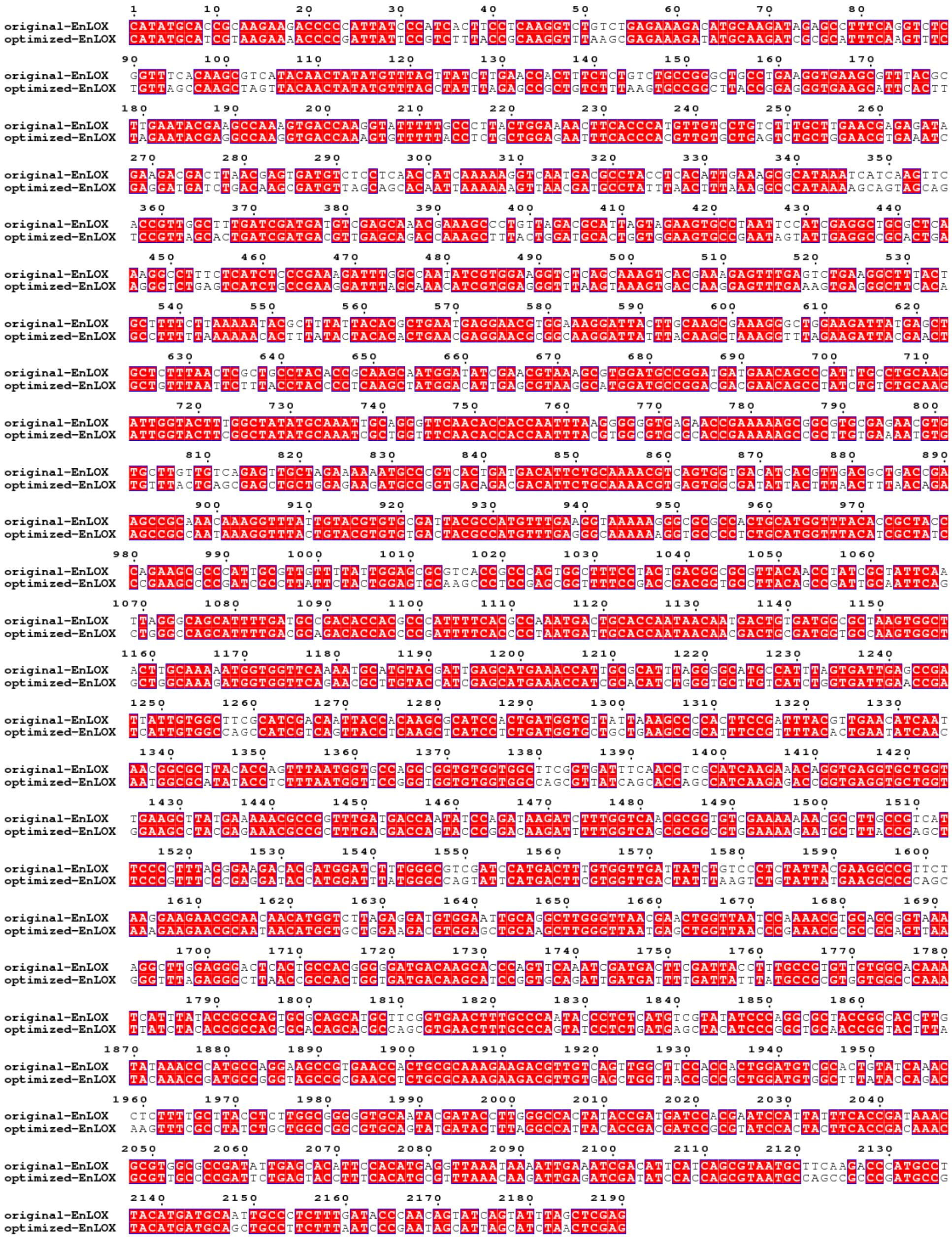

Figure S1. The nucleic acid sequences alignment of original and codon-optimized EnLOX.

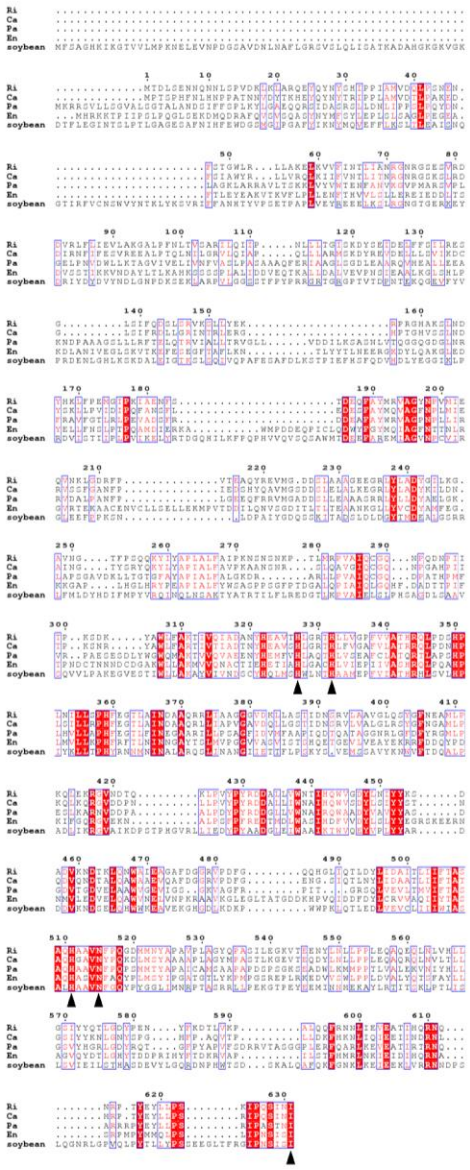

Figure S2. Multiple sequences alignment of LOXs.

Residues in red are conserved in all sequences in the alignment, active sites are pointed by black triangles.

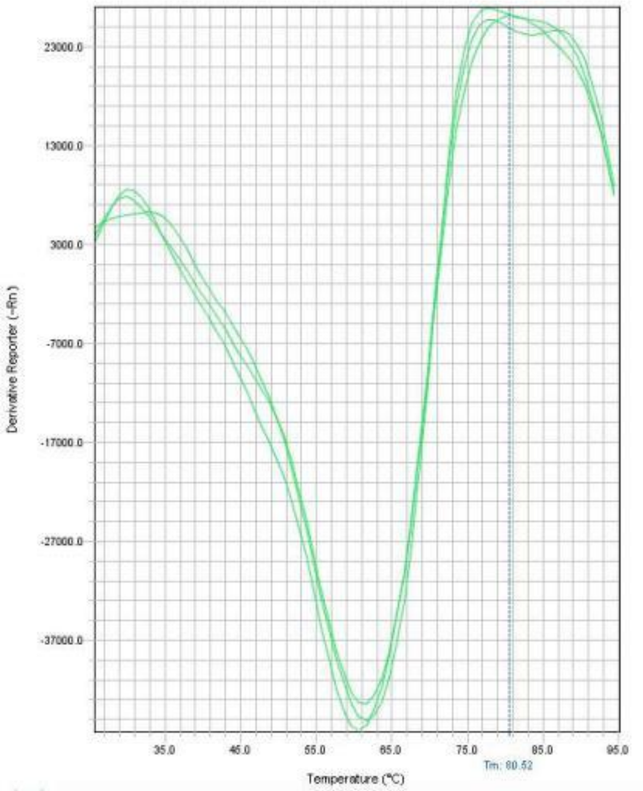

Figure S3. Melt curves of EnLOX in Thermal shift assay.

Table S1. Biochemical properties of EnLOX and LOXs from various sources.

Enzyme	M.W (kDa)	Specific activity (U/mg)	Optimum pH	ptimum temperature (°C)	Half-life	K_m (µmol L^{-1})	K_{cat} (s^{-1})	K_{cat}/K_m (L µmol^{-1} s^{-1})	Reference
E. norvegicus LOX	70	40.34	7.5	50	6 h (50°C)<br>9 h (40°C)	3.64	16.48	4.53	This study
Calothrix LOX	71	73.1	8	25	8.14 h (40°C)	6.7	93.2	13.85	16
Rivularia LOX	71	68.8	8.5	30	8.14 h (40°C)	7.5	80.6	10.79	16
T. bouteillei LOX	71	10.1	8	30	23.7 h (40°C)	16.5	12.8	0.78	16
Anabaena LOX	70	10.4	9	35	2.3 min (50°C)	56	30.3	0.54	14, 19
M. xanthus LOX	80	24	3	30	7.1 min (50°C)	380	9.2	0.02	1, 34
P. aeruginosa LOX	70	28.5	7.5	25	10 min(50°C)	48.9	23.5	0.48	18
B. thailandensis LOX	75	26.4	7.5	25	21 min (50°C)	41.5	93.7	2.26	11
A. aegerita LOX	80	51.34	7.5	25	NR	295.5	103.9	0.35	32
N.benthamiana LOX	97.4	0.35	6	35	NR	3.9	NR	NR	26
Pleurotus LOX	72.3	130.3	7	35	NR	40.3	157	3.89	36
Cyanothece LOX	65	NR	9.3	NR	NR	18	30.7	1.7	45
P. ostreatus LOX	67	33	8	25	5 min (45°C)	130	25.7	0.2	36
Soybean LOX	94	105.7	9	15	30 min (45°C)	150	NR	0.17	35, 41
